# How to run a successful Journal

**DOI:** 10.12669/pjms.336.14097

**Published:** 2017

**Authors:** Shaukat Ali Jawaid, Masood Jawaid

**Affiliations:** 1 Shaukat Ali Jawaid* Chief Editor, Pakistan Journal of Medical Sciences, Karachi - Pakistan; 2 Masood Jawaid Associate Editor Pakistan Journal of Medical Sciences, Karachi - Pakistan

**Keywords:** Publishing, Biomedical Journals, Logistics and Technical issues

## Abstract

Publishing and successfully running a good quality peer reviewed biomedical scientific journal is not an easy task. Some of the pre-requisites include a competent experienced editor supported by a team. Long term sustainability of a journal will depend on good quality manuscripts, active editorial board, good quality of reviewers, workable business model to ensure financial support, increased visibility which will ensure increased submissions, indexation in various important databases, online availability and easy to use website. This manuscript outlines the logistics and technical issues which need to be resolved before starting a new journal and ensuring sustainability of a good quality peer reviewed journal.

## INTRODUCTION

A Journal is only as good as its Editorial Team and the quality of its reviewers. Hence for the successful running of a journal the first and the foremost important pre-requisite is a competent editor supported by a team of editorial staff. In fact, there are some bare minimum requirements which are essential for running a successful journal and ensure its sustainability in the long run. These are as under:

1. Good quality manuscripts
2. Proper office and Minimum essential staff
3. Financial sustainability
4. Active Editorial Board
5. Good Quality of Reviewers
6. Increased visibility
7. Indexation in various databases
8. Online availability
9. Easy to use Website

## HOW TO GET STARTED

After having completed the legal requirements and governmental formalities of obtaining a declaration, one should first assess the strength and resources because everything which follows will depend on that. It is always important to move slowly and ensure steady progress. Decide about the frequency of your journal/publication which could be annual, biannual, quarterly, bimonthly or monthly. Be careful in selecting the members of the editorial board and founder members. Do not go for the big names that are too busy and do not have time but instead select those who are keen and interested. Decision about the frequency of publication will be determined keeping in view the financial resources. Of course timely publication as per frequency is extremely important.

When starting a new journal, there are some logistic and technical issues which have to be resolved. First of all it is essential that there should be a proper office with minimum full time staff. This also includes better title, designing a website with good impact, deciding about some manuscript management system, applying for ISSN number and in case of both print and online versions; one will need separate ISSN numbers for both. Decide about the Abbreviation of the Journal which should appear everywhere and which will be used while writing references.^1^ In the current world of online publishing now it’s not a choice but important quality yardstick to have system to issue DOI (digital object identifier) to every manuscript which you publish.^2^

### Peer Review System

Decision about Peer Review system which one has to be followed i.e. single blind, double blind or Open Peer Review System. It may take some time to develop a good Reviewers Database for the journal and it is up to the Editorial team to ensure to grow and retain good reviewers.^3^ It is also important that comments by the Reviewers are reviewed by the Editorial staff removing any harsh, inappropriate words from the review before they are communicated to the authors. 4

### CONTENTS OF THE JOURNAL

The well known functions of the media, print or electronic are to inform, educate and guide. Hence the contents of a scientific journal can reflect most of the above functions. One can include stories and manuscripts which have useful information for the readers of the journal. Secondly the journal can serve as a useful medium to educate the general public or its intended readership on various issues of national importance keeping in view the local socio economic development, environment and cultural values. To do this it is important that the writer is intelligent and competent enough to educate the readership after having compiled all the essential information. In this the writer/contributor is required to interpret the events, happenings and highlight their likely effects or repercussions. The third and the most important function is guide which cannot be accomplished unless the writer has kept himself/herself informed, fully abreast of the latest developments on the subject he or she is writing. An ignorant author is no less than a weapon of mass destruction because instead of guiding the readers, he or she can misguide the public and the readers at large. In order to ensure that no wrong messages is communicated, the concept of peer review was coined many years ago whereby the write-ups before publication are looked into by experienced, competent individuals in the respective field apart from the Editor. It is the job of the Editor to arrange and organize an effective peer review system for the journal. While peer review has its own advantages it also has some disadvantages as it can slow down the pace of work, can result in delay in publications and at times even may not be able to detect any wrong information or scientific, academic misconduct.

### VARIETY OF CONTENTS

The journal must have a wide variety of contents to satisfy the needs of every class and group of readers. Starting from original research, the contents can include Reviews, Clinical Case Series, Short/Brief Communications, short stories, important news, Book Reviews, Obituary Notes, Quiz etc., while comments from the readers should be encouraged and welcomed. Conference Reports could be quite useful and informative for many who for some reasons cannot attend these meetings. Information about the forthcoming important events, conferences, seminars, symposia could also be very useful addition and informative for the readers.

A good review should help the authors to revise and improve the quality of their manuscript. Committee on Publication Ethics (COPE) has prepared some Flowcharts which provide useful guidelines to the editors to handle scientific misconduct.^5^

## ICMJE GUIDELINES

International Committee of Medical Journal Editors (ICMJE) has formulated some guidelines for submission of manuscripts to biomedical journals which are revised after every couple of years.^6^ Biomedical journals the world over have a standard format. The important components of the original, the most published manuscripts are covered in **SIMRAD** wherein

**S** stands for structured abstract.

**I** stand for Introduction

**M** stands for Methods

**A** stands for analysis

**D** stands for Discussion.

The manuscript is then followed by conclusions, acknowledgements and the relevant references. Authors are required to declare their conflict of interest and the source of Funding if any. Structured abstract usually contains four sub-headings i.e. Objective, Methods, Results and Conclusion followed by relevant Key Words. For Case Reports, Special Communications a one paragraph Summary is considered enough. However, while writing a Review Article, the International Committee of Medical Journal Editors (ICMJE) guidelines require that the summary must also provide information about the Data Bases which were used for literature search and the time period, how the information was extracted.^6^

For Journals/publications covering Culture and Sociology, it is important that they include manuscripts which carry some message relevant in these fields. Success stories which could encourage others to follow the same footsteps could be a useful addition to the contents. Real life events which have some message how to tackle some particular problems would be welcomed by the readers. Contents should be such that it invites the readers and when the readership increases, it is considered a very healthy sign for the publication.

### Instructions for Authors

The print issue as well as the journal website must contain comprehensive information for the authors. It should give details about the maximum number of words which the journal will accept in different categories of write-ups, maximum number of figures and tables to be included. It should provide comprehensive guidelines to the authors including journal policy regarding plagiarism.

### Ensuring Transparency

Details about processing fee if any, publication charges, peer review system, types of manuscripts accepted for publication, details about fast track processing of manuscripts, Registration of trials, pharma industry sponsored studies, likely time period from submission to peer review etc., must be clearly mentioned in the instructions ensuring transparency. There should be no hidden charges. It is also advisable that the journal provide some forum for redress of grievances, complaints to the authors, readers, reviewers, advertisers. Those whose manuscripts are rejected should also have a chance to appeal against the decision. These days some journals have appointed Ombudsman for this and their contact details are published in the journal as well as on the journal website.

### Copy Editing

Sometimes the manuscript received for publication need improvement of English language and Grammar. If the journals can afford, they should appoint Copy Editors. They are supposed to not only improve the English language and Grammar but also eliminate repetition of information, redrawing the tables and figures if need be so that the message is conveyed effectively. However, if there is no copy editor in the staff, this has to be done by the Editor. Some software’s i.e. Grammarly.org are also available for improvement of English language and Grammar but one has to subscribe it.^7^

## PRINT AND ONLINE ISSUES

While print version of the journal has its importance as seeing is believing, but it is expensive and takes time. On the other hand, Online Edition of the Journal is not only highly economical but it is also very quick. Hence it is essential that a journal/publication should have both versions, print format as well as the Online Edition which will increase the visibility of the publication manifold.

## FINANCIAL SUSTAINABILITY

It is one of the most important aspects and the Editor should ensure adequate funding to sustain the long term publication. The sources of funding could be through donations, grants from philanthropists, Trusts or Foundations, Government Grants if possible. Subscription generation takes time and even then it does not fetch much funding for sustained growth. However, an important source of income could be through advertisements for which one needs a strong committed business team. Initial few years could be very taxing but as the time passes and the journal/publication earns some credibility, readership and improved visibility, things start becoming a bit easier. Once the Journal gets established, “Authors Pay Business Model” ensures financial sustainability. However, even then there are too many problems which one has to face. It must be remembered that running and managing a good quality peer reviewed journal in any subject, specialty particularly in the Developing world is a very frustrating and stressful job because of financial constraints and lack of trained, experienced human resource. While training the staff will take time but how to retain them once they have been trained is yet another uphill task for the management.^8^’^9^

### Recognition by Regulatory Agencies

Recognition by the regulatory agencies for example in case of Pakistan by Pakistan Medical & Dental Council (PM&DC) and Higher Education Commission (HEC) of Pakistan will ensure more submissions. Sometimes HEC also provide some financial grants to the journals but the journals have to provide the audited accounts of this grant at the end of the year.

### Indexation in various Databases

Recognition and coverage in Directory of Open Access Journals (DOAJ), inclusion in Medline PubMed, PubMed Central, Scopus, recognition by Thompson Reuter (Clarivate Analytics (https://clarivate.com/) and getting Impact Factor, applying for DOI numbers after contract with CrossRef all ensure credibility, increased visibility and readership which in turn results in increased submissions. However, all this may not be easy as most of these databases have Evaluation Committees to look at the quality of contents before accepting to recognize the journal. Moreover, timely regular publication is a pre-requisite.^10^

### Plagiarism Check

A number of free software’s are available on the net to check plagiarism but the most important one is Turnitin/iThenticate which one has to subscribe.^11^ In Pakistan Higher Education Commission (HEC) has provided this facility of checking plagiarism through TurnitIn to all the public sector universities including medical institutions for their faculty members. All accepted manuscripts must be screened for plagiarism before they are accepted for publication to avoid embarrassment later and avoid retractions. Software’s to check English language and Grammar like Grammarely.org are also available but again one has to subscribe all this.^7^

## ISSUES RELATED TO OWNERSHIP

The problems and difficulties related to running a successful journal are also related to the ownership of these publications. If the Society, Organization, Foundation or Trust or the institutions including Universities are the owners, they can set aside enough funding for this project. Not only that, they can even acquire the services of trained and experienced people to run and manage the journal. However, while assuring financial support, these institutions, organizations must also ensure that the Editor has enough independence to take day to day decisions related to the journal which are extremely important. But if the Editor has to take permission for each and every action, decision, from the higher authorities, it will not only slow down the whole publication process but never encourage the Editorial team to take initiative. Ideally the editors should have a contract appointment wherein their tenure, rights and responsibilities should be clearly stated.

During the past two decades, there have been tremendous developments in Information technology which on one hand has made the job of Editors easy but at the same time, it has also put them under lot of stress and it has become quite difficult for the Editors to come upto the expectations of authors who are quiet impatient, wish to see their manuscripts in print as soon as possible. They are also known to be the most dangerous pressure groups, which the editors have to face.^12^

In conclusion, running and managing a good quality journal faced with financial and human resource constraints is not easy. It is quite frustrating and stressful job and all these problems, difficulties which one can encounter must be looked into before embarking on this journey of publishing a Journal. However, professional competencies of the Editor will ensure smooth sailing to a great extent.^13^
